# Expression mode and prognostic value of FXYD family members in colon cancer

**DOI:** 10.18632/aging.203290

**Published:** 2021-07-15

**Authors:** Ming Jin, Hui Zhang, Jun Yang, Zhen Zheng, Kaitai Liu

**Affiliations:** 1 The Affiliated Lihuili Hospital, Ningbo University, Ningbo, China; 2 Department of Radiation Oncology, The Affiliated Lihuili Hospital, Ningbo University, Ningbo, China; 3 Ningbo Diagnostic Pathology Center, Ningbo, China

**Keywords:** FXYD family, colon cancer, The Cancer Genome Atlas, prognosis, survival

## Abstract

The FXYD gene family comprises seven members that encode a class of small-membrane proteins characterized by an FXYD motif and interact with Na^+^/K^+^-ATPase. Until now, the expression patterns and prognostic roles of the FXYD family in colon cancer (CC) have not been systematically reported. Gene expression, methylation, clinicopathological features and the prognoses of CC patients were obtained from The Cancer Genome Atlas (TCGA) database. The expression feature and prognostic values of FXYD members were identified. Gene set enrichment analysis (GSEA) was performed to explore the potential mechanism underlying the function of the FXYD family in CC. Tumor Immune Estimation Resource (TIMER) and CIBERSORT analysis were used to assess the correlations between FXYD family members and tumor immune infiltrating cells (TIICs). FXYD family members were differentially expressed in CC except for FXYD2. FXYD2, FXYD3 and FXYD4 were revealed as independent prognostic factors for recurrence, while FXYD3 and FXYD7 were identified as prognostic factors for survival according to univariate and multivariate analyses with Cox regression. GSEA revealed that FXYD family members were involved in complicated biological functions underlying cancer progression. TIMER and CIBERSORT analyses showed significant associations between FXYD family genes and TIICs. The present study comprehensively revealed the expression mode and prognostic value of FXYD members in CC, providing insights for further study of the FXYD family as potential clinical biomarkers in CC.

## INTRODUCTION

According to the latest data, colon cancer (CC) is one of most frequently diagnosed cancers, and it is predicted to be a leading cause of cancer death worldwide in 2021 [1]. Colon cancer is also one of the most common malignant tumors of the digestive tract in China [2]. Approximately one-fourth of colon cancer patients have distant metastases at first diagnosis, resulting in a poor prognosis [3]. Although modern therapies extend the survival time, the prognosis of CC remains frustrating, with a 5-year survival rate of 30-40%, even in patients who undergo curative resection after systemic therapy [4, 5]. Therefore, the exploration of novel molecular markers has crucial clinical significance for improving the diagnosis and treatment of colon cancer.

Abnormal DNA methylation is a major early promoter to CC development. Previous study has showed that aberrant methylation in DNA regulatory regions could upregulate oncogenes and downregulate tumor suppressor genes. Liang et al. found some methylation-regulated differentially expressed genes play an important role in colon cancer progression [6]. Wang et al. reported that hypomethylated and hypermethylated differentially methylated CpG sites could be used as diagnostic and prognostic biomarkers in CC [7]. The FXYD gene family members were first defined in 2000 as small ion transport regulators or channels with consensus sequences [8]. They are small membrane proteins that share an FXYD motif beginning with the FXYD sequence: that is, phenylalanine (F), X, tyrosine (Y), and aspartate (D). In addition, FXYD family members all have two conserved glycine residues and a serine residue in their transmembrane domains [9, 10]. The FXYD gene family contains seven members, FXYD1, FXYD2, FXYD3, FXYD4, FXYD5, FXYD6 and FXYD7, that function in Na^+^/K^+^-ATPase transport by modulating transporter properties [11]. Na^+^/K^+^-ATPase is intimately associated with the epithelial-to-mesenchymal transition (EMT) and TGF-β1 and NF-κB pathways in malignant tumors [12, 13]. In recent years, studies have reported that FXYD family members play important roles in tumor progression, including esophageal carcinoma, rectal cancer, hepatocellular carcinoma, pancreatic cancer, lung cancer and ovarian cancer [14–21]. Research showed that high expression of FXYD3 in esophageal carcinoma promoted tumor progression, resulted in an unfavorable prognosis [14]. High expression of FXYD3 increased incidence of distant metastasis after undergoing preoperative radiotherapy (RT) in patients with rectal cancer [15]. Tamura M et al. found that FXYD5 might be an independent predictor of survival for patients with non-small cell lung cancer [22]. Studies revealed that patients with high FXYD5 expression might benefit less from RT compared to these with low FXYD expression in head and neck cancer [23, 24]. However, systematic investigation on the features and functions of the entire FXYD gene family in specific cancers has not yet been well reported. In the present study, we comprehensively explored the whole expression picture and prognostic value of the entire FXYD gene family in colon cancer by analyzing the data from The Cancer Genome Atlas (TCGA).

## RESULTS

### Differential expression of FXYD family member genes in CC

**Figure 1:**
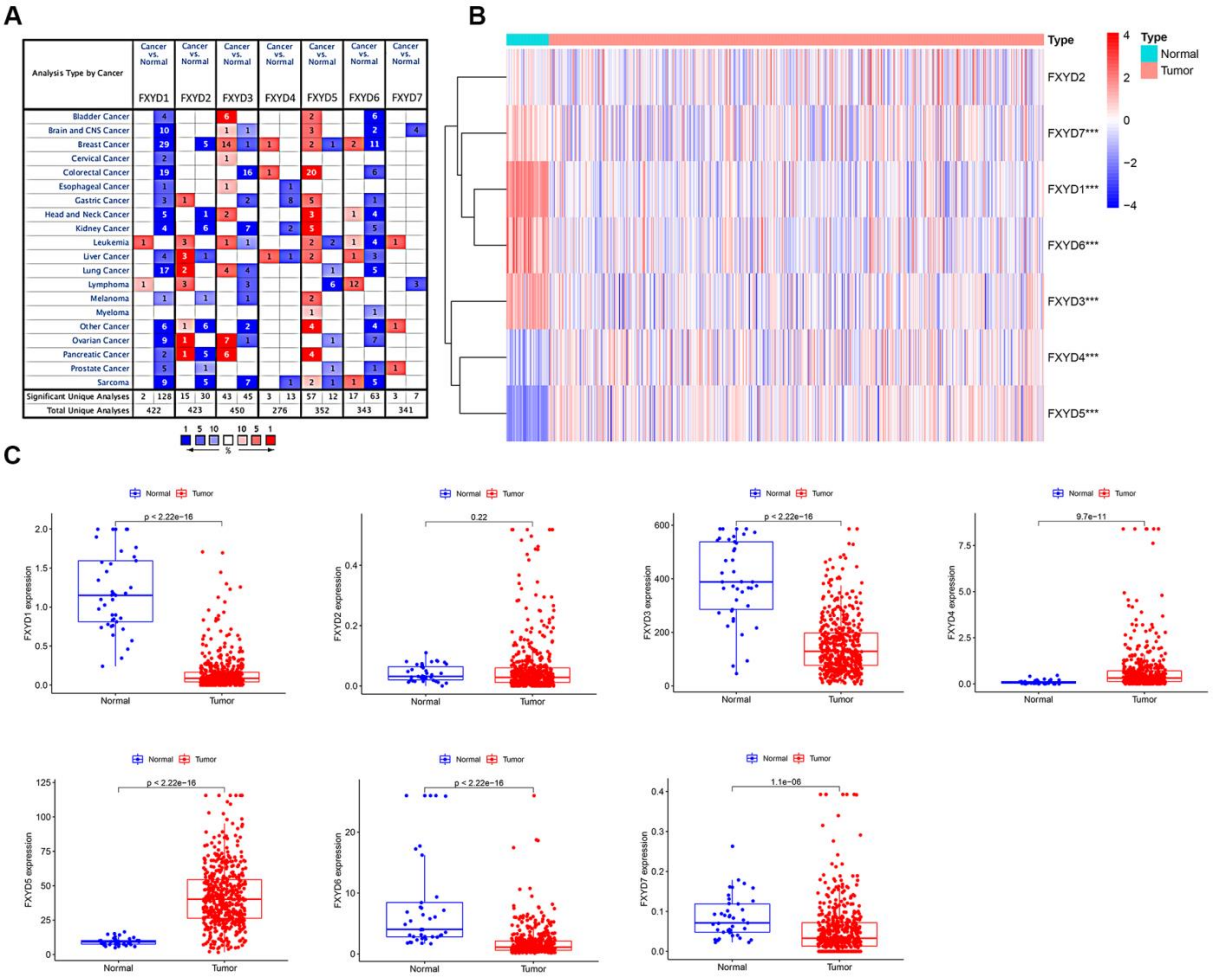
(**A**) The expression heatmap of FXYD family genes in different types of cancers. Red and blue indicate the numbers of datasets with statistically significant (P<0.05) increased and decreased levels of FXYD family members, respectively. (**B**) Differential expression of FXYD family genes between the CC samples and normal tissues represented by a heatmap. The tree diagram at the left showed the cluster analysis between FXYD family members. ***, *P*<0.001. (**C**) Differential expression of FXYD family genes between the CC samples and normal tissues represented by box plots.

The apparently different gene expression levels of FXYD family members in various types of cancer samples and matched normal tissues, as obtained from the ONCOMINE database, is shown in Figure 1A. Regarding colorectal cancer, FXYD1, FXYD3, and FXYD6 were significantly downregulated in the cancer samples, while FXYD4 and FXYD5 were overexpressed (*P*<0.05, fold change>1.5). Subsequently, we used the TCGA database, which included 480 CC samples and 41 adjacent normal samples, to assess the expression picture of FXYD family members. The heatmap showed the differential expression of FXYD family member genes between the CC samples and normal tissues (Figure 1B). Moreover, the box plot demonstrated that six of seven members presented aberrant expression levels in CC, with FXYD2 being the exception. FXYD1, FXYD3, FXYD6 and FXYD7 were significantly downregulated in the CC samples, while FXYD4 and FXYD5 were both obviously increased compared to normal tissues, which was consistent with the results obtained with the ONCOMINE data (Figure 1C). We further assessed the association between expression of FXYD family members and clinicopathological characteristics (Supplementary Tables 1–7). Results showed that the expression of FXYD4 was significantly related with gender. The expression of FXYD6 was significantly related with age. High expression of FXYD2 and FXYD5 were associated with more advanced T stage. The expression of FXYD1, FXYD3 and FXYD6 were markedly correlated with N stage.

### Methylation of the promoter regions of FXYD genes in CC

**Figure 2:**
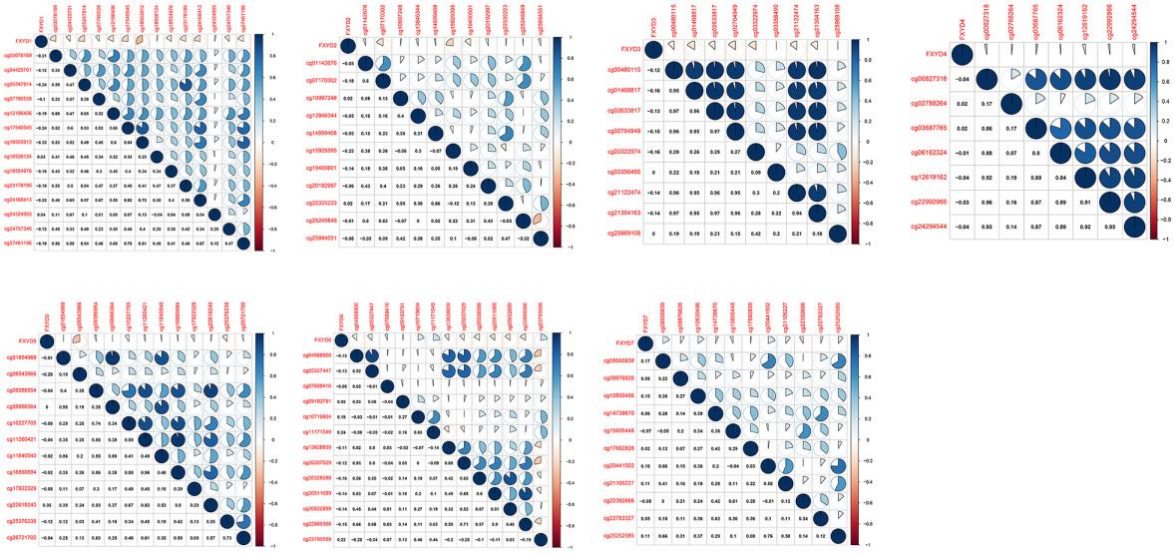
Correlation between methylation levels and expression of FXYD family members in CC.

DNA methylation is one of the most common epigenetic events that results in abnormal gene expression in cancer. We analyzed the methylation levels of cg sites in the promoter areas and assessed the association between methylation and expression of FXYD members (Figure 2). Pearson correlation analyses showed that the expression of FXYD family members was negatively associated with methylation levels, especially for FXYD1, FXYD3, FXYD5 and FXYD6. The results indicated that abnormal methylation might account for the aberrant expression of these genes.

### Prognostic value of FXYD family members in CC

**Figure 3:**
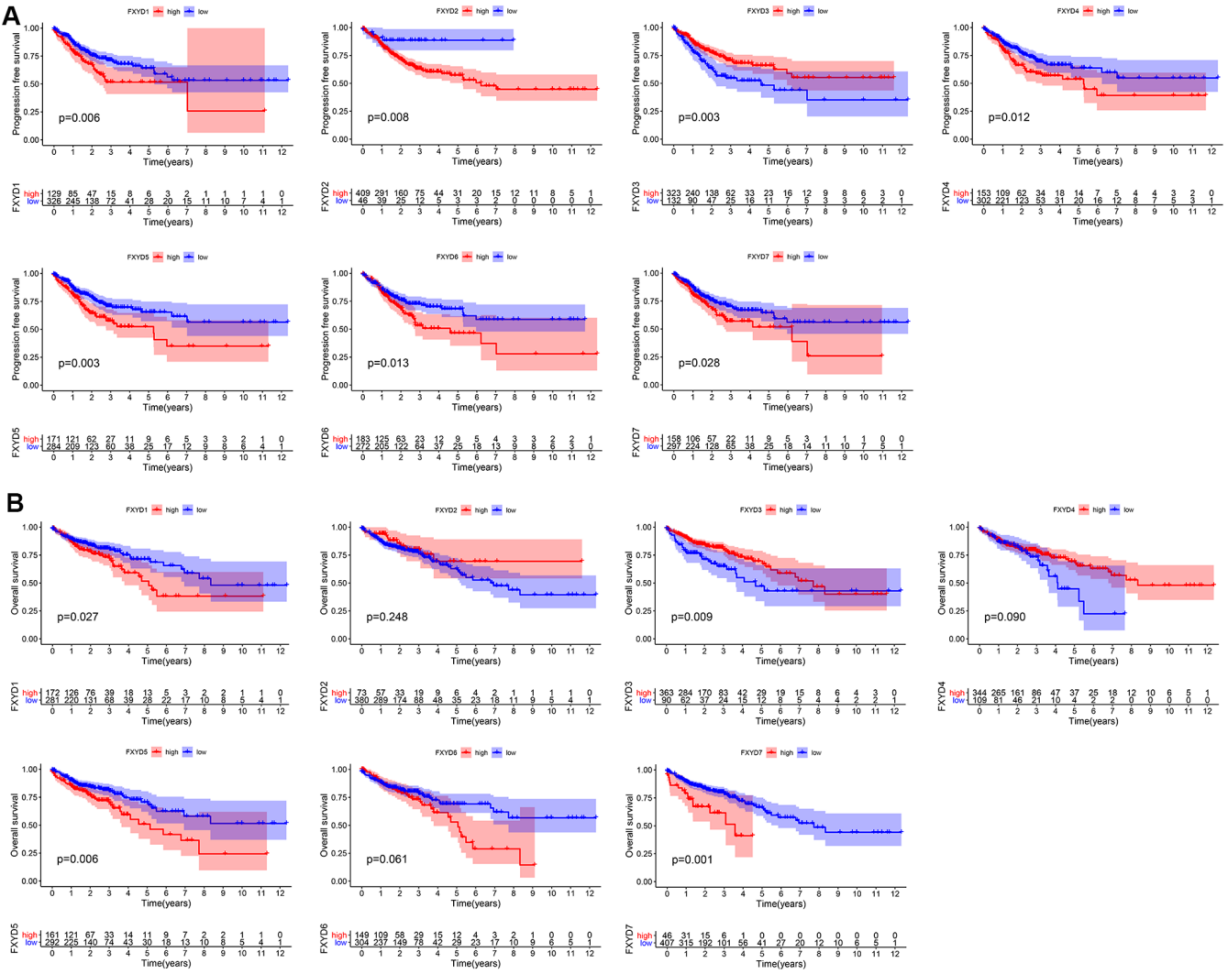
**Prognostic value of FXYD family members in CC.** (**A**) Recurrence outcomes and (**B**) Survival outcomes.

**Figure 4:**
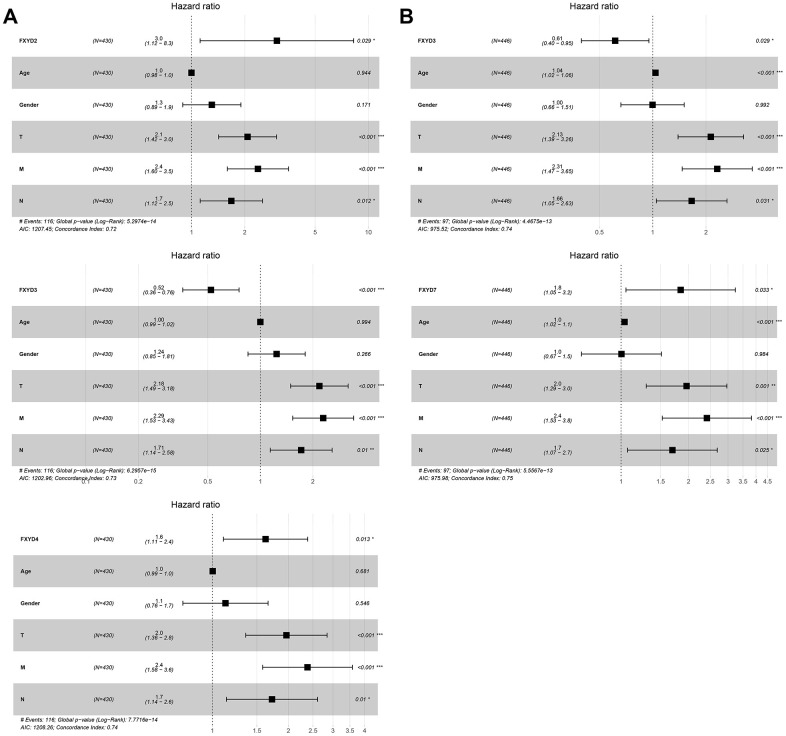
**The results of multivariate Cox regression analyses of significant prognostic factors represented by forest plots.** (**A**) Recurrence outcomes and (**B**) Survival outcomes. *, *P*<0.05. **, *P*<0.01. ***, *P*<0.001.

Furthermore, we evaluated the prognostic effects of FXYD members in CC. Kaplan-Meier analyses indicated that all members had a significance effect in terms of recurrence outcomes, and four members showed predictive values for survival outcomes: FXYD1, FXYD3, FXYD5 and FXYD7 (Figure 3). In addition, we assessed the prognostic value of the clinicopathologic characteristics and the FXYD family members by Cox proportional hazards regression. With respect to recurrence, T stage, N stage, M stage and FXYD1-6 were identified as significant predictive factors in the univariate analysis (*P*<0.05) (Table 1). When a multivariate analysis was performed, we identified FXYD2, FXYD3 and FXYD4 as independent prognostic factors (*P*<0.05) (Figure 4A). In regard to survival, age, T stage, N stage, M stage, FXYD1, FXYD3, FXYD5 and FXYD7 were identified as significant prognostic factors according to the univariate analysis (*P*<0.05) (Table 2). FXYD3 and FXYD7 were further confirmed as independent prognostic factors based on the results of the multivariate analysis (Figure 4B).

**Table 1:** Univariate Cox regression analysis of FXYD members expression as recurrence predictors.

**Variable**	**Univariate analysis**		
	**Hazard ratio**	**95% CI**	***P* value**
Age	0.997	0.982-1.012	0.683
Gender	1.229	0.849-1.799	0.274
T stage	2.863	2.009-4.081	<0.001*
N stage	2.664	1.834-3.871	<0.001*
M stage	3.268	2.255-4.736	<0.001*
FXYD1 expression	1.560	1.062-2.291	0.023*
FXYD2 expression	3.611	1.332-9.792	0.012*
FXYD3 expression	0.525	0.363-0.760	<0.001*
FXYD4 expression	1.673	1.160-2.413	0.006*
FXYD5 expression	1.692	1.174-2.437	0.005*
FXYD6 expression	1.644	1.140-2.372	0.008*
FXYD7 expression	1.443	0.993-2.095	0.054

*, P<0.05.

**Table 2:** Univariate Cox regression analysis of FXYD members expression as survival predictors.

**Variable**	**Univariate analysis**		
	**Hazard ratio**	**95% CI**	***P* value**
Age	1.029	1.011-1.047	0.002*
Gender	1.171	0.783-1.752	0.442
T stage	2.638	1.774-3.921	<0.001*
N stage	2.419	1.609-3.638	<0.001*
M stage	3.263	2.168-4.912	<0.001*
FXYD1 expression	1.568	1.049-2.344	0.028*
FXYD2 expression	0.719	0.393-1.317	0.285
FXYD3 expression	0.579	0.376-0.894	0.014*
FXYD4 expression	0.692	0.442-1.085	0.109
FXYD5 expression	1.756	1.177-2.621	0.006*
FXYD6 expression	1.493	0.991-2.248	0.055
FXYD7 expression	2.322	1.328-4.062	0.003*

*, P<0.05.

### Potential molecular mechanism underlying the roles of prognostic FXYD family members in CC

**Figure 5:**
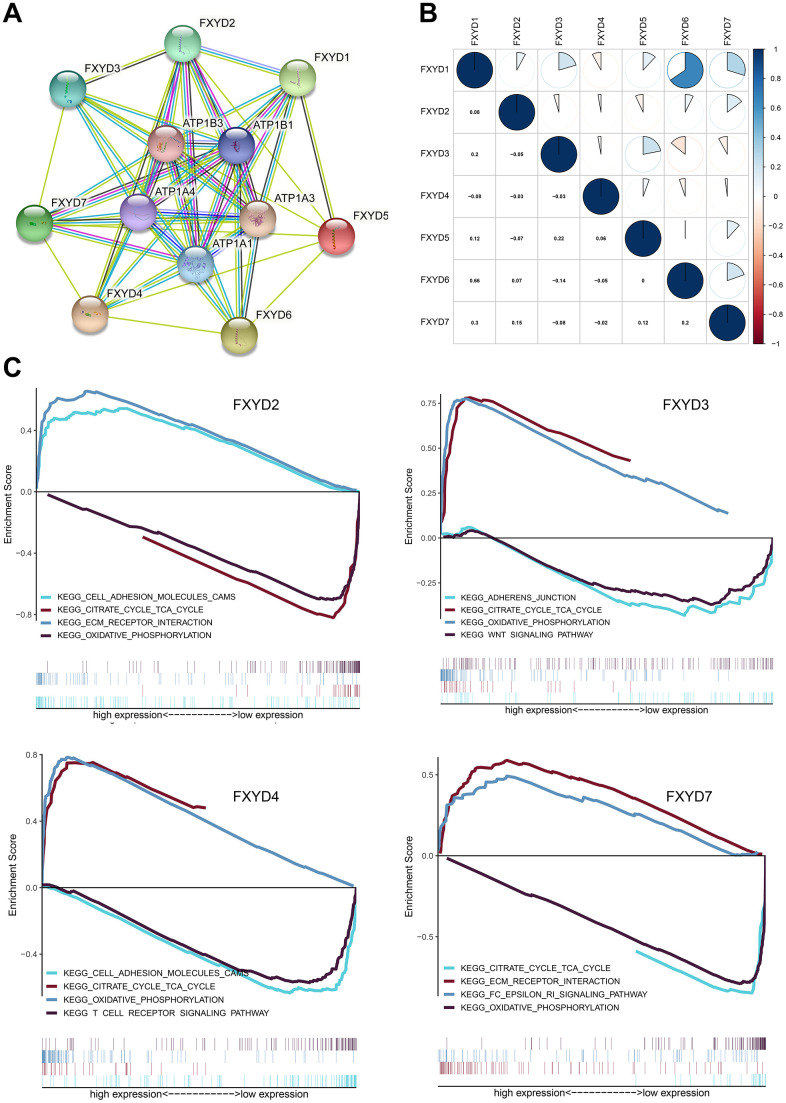
(**A**) Protein–protein interaction network among FXYD gene family members. (**B**) Correlations between FXYD family genes. (**C**) Kyoto Encyclopedia of Genes and Genomes (KEGG) enriched pathways associated with FXYD2, FXYD3, FXYD4 and FXYD7 in GSEA.

To identify the potentially related proteins interaction and evaluate whether the FXYD family genes were correlated with each other, we constructed a PPI network by STRING database and performed a Pearson correlation based on the gene expression data from TCGA. Our study found that the FXYD gene family had a strong correlation with the Na^+^/K^+^-ATPase subunit (Figure 5A). The FXYD1 gene was significantly correlated with FXYD3, FXYD6 and FXYD7. The FXYD3 gene was significantly correlated with FXYD5. The FXYD6 gene was significantly correlated with FXYD7 (Figure 5B). Besides, we performed gene set enrichment analysis (GSEA) to explore the potential molecular mechanism underlying the prognostic effects of FXYD family members in CC. The results indicated that high expression of FXYD2 was positively related to “ECM receptor interaction”, “cell adhesion molecules CAMs” and negatively related to “citrate cycle” and “oxidative phosphorylation”. High expression of FXYD3 was positively related to “citrate cycle”, “oxidative phosphorylation” and negatively related to “adherens junction”, “Wnt signaling pathway”. High expression of FXYD4 was positively related to “oxidative phosphorylation”, “citrate cycle” and negatively related to “T cell receptor signaling pathway”, “adhesion molecule CAMs”. High expression of FXYD7 was positively related to “FC epsilon RI signaling pathway”, “ECM receptor interaction” and negatively related to “oxidative phosphorylation”, “citrate cycle” (Figure 5C). Remarkably, all of these genes were significantly related to oxidative phosphorylation and the citrate cycle, which supported their interactions with Na^+^/K^+^-ATPase.

### Associations between TIICs and the FXYD family in CC

**Figure 6:**
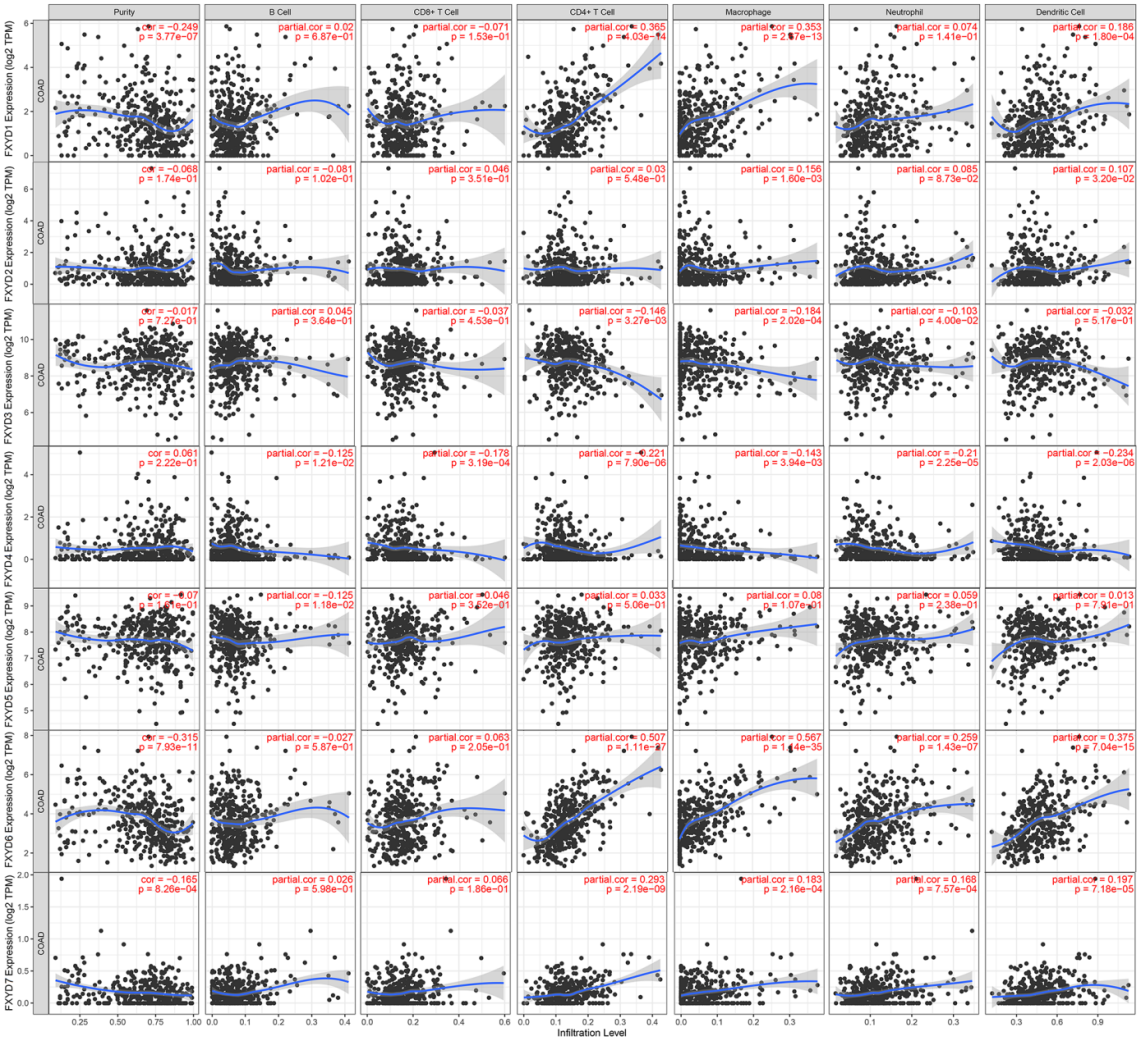
Correlations between tumor infiltrating immune cells and independently prognostic FXYD family genes (FXYD2, FXYD3, FXYD4, and FXYD7).

**Figure 7:**
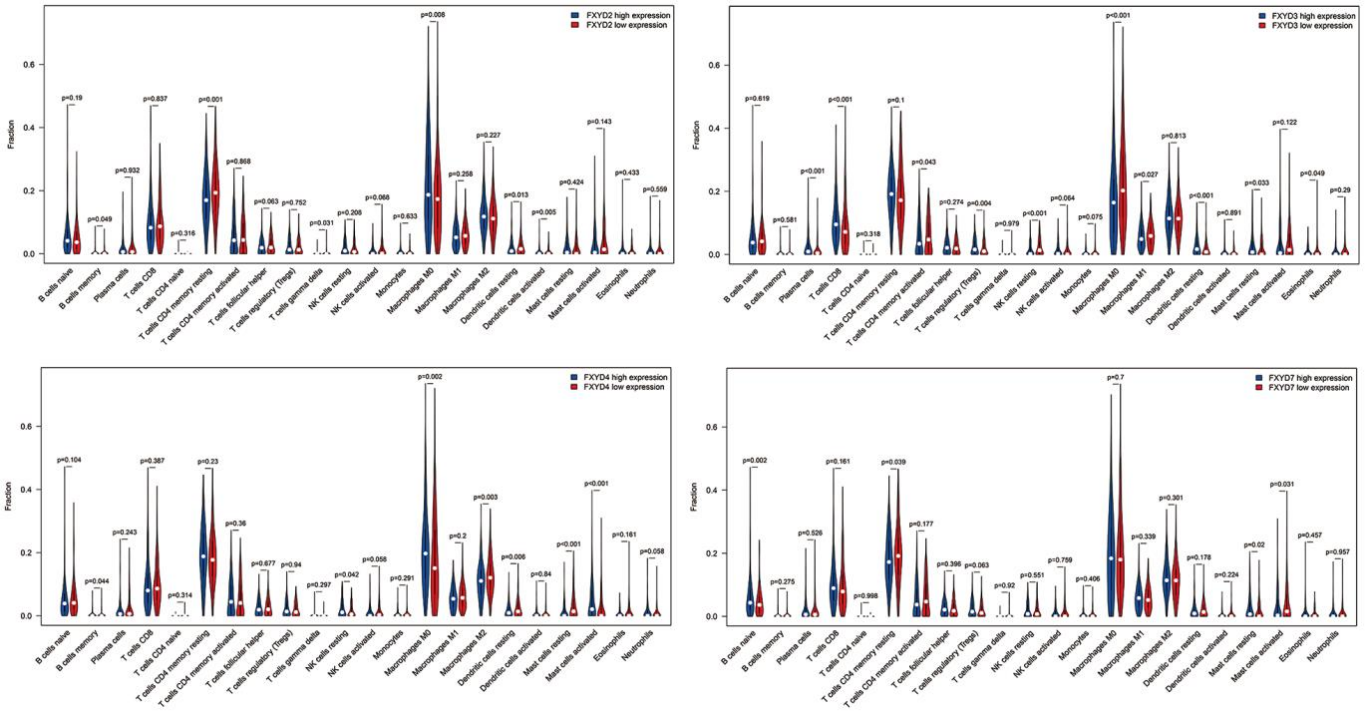
The differentially tumor infiltrating immune cells between high expression group and low expression group in FXYD2, FXYD3, FXYD4 and FXYD7.

In recent years, researchers have paid increasing attention to the relationship between the immune microenvironment and cancer progression. Therefore, we assessed the correlations between FXYD members and TIICs (Figure 6). Analysis of the TIMER database demonstrated that FXYD1, FXYD6 and FXYD7 had significantly negative associations with tumor purity, while these genes had markedly positive correlations with CD4^+^ T cells, macrophages and dendritic cells. The FXYD3 had a significantly negative association with CD4^+^ T cells, macrophages and neutrophils, and the FXYD4 had an observably negative correlation with all TIIC types. FXYD6 and FXYD7 were significantly positively correlated with CD4^+^ T cells, macrophages, neutrophils and dendritic cells. CIBERSORT results showed the relationship between FXYD family genes and the 22 immune cell types (Figure 7). High expression of FXYD2 was significantly associated with more memory B cell, M0 macrophages, activated dendritic cells, and less resting memory CD4^+^ T cells, resting dendritic cells. High FXYD3 expression was associated with more plasma cells, CD8^+^ T cells, regulatory T cells (Tregs), resting dendritic cells, resting mast cells, and less activated memory CD4^+^ T cells, M0 macrophages, M1 macrophages. High FXYD4 expression was positively related with resting NK cells, M0 macrophages, activated mast cells, and negatively related with M2 macrophages, resting dendritic cells. High expression of FXYD2 was positively related with naive B cells, resting mast cells and negatively related with resting memory CD4^+^ T cells, activated mast cells. Our results suggested that FXYD family members may function as regulators of the immune microenvironment in CC, which is worthy of further investigation.

## DISCUSSION

The FXYD family was initially defined by Sweadner and Rael because of their consensus sequences [8]. They all share an FXYD motif, two conserved glycine residues and a serine residue in their transmembrane region [9, 10]. According to recent studies, FXYD family members are mainly involved in the regulation of Na^+^/K^+^-ATPase modulation and may participate in tumor progression, particularly in TGF-β1-mediated EMT targeting of Na^+^/K^+^-ATPase [12, 13]. However, no systematic report has provided an overview of the whole FXYD gene family in CC. To our knowledge, the present study is the first to comprehensively investigate the expression modes of the FXYD family and to systematically illustrate the correlations between FXYD family genes and the prognosis of patients with CC, offering further suggestions for the clinical value of FXYD family genes in CC.

In recent years, researchers have found that FXYD2 shows different expression patterns in different kinds of renal cell carcinoma as defined by immunohistochemistry [25]. FXYD3 was found to be upregulated in several cancers, such as hepatocellular carcinoma, pancreatic cancer, endometrial cancer and breast cancer [16, 18, 26, 27]. However, the expression feature of FXYD3 in colorectal cancer is controversial. Anderle and Widegren [28, 29] found that there was no significant change of FXYD3 expression level in colon cell lines or tissues compared to the control groups. In contrast, Even Kayed [18] reported that FXYD3 was expressed at lower levels in colon tissues, which is consistent with our results. FXYD5 was found to be upregulated in endometrial cancer, and patients with high tumor grades tended to have higher expression levels of FXYD5 [13]. FXYD6 was identified as an oncogenic factor in osteosarcoma cells with higher expression levels than in normal tissues [30, 31].

Our systematic results showed that FXYD family members were differentially expressed in CC except FXYD2. FXYD1, FXYD3, FXYD6 and FXYD7 were significantly downregulated in cancer samples, while FXYD4 and FXYD5 were markedly overexpressed. To explore the underlying reason for the aberrant expression of the FXYD family in CC, we further analyzed the methylation levels in promoter areas. Methylation and demethylation of cg sites in promoter regions can results in silencing and reactivation of genes, by which genes take effect in cell proliferation, apoptosis, and cell cycle [32, 33]. Methylation changes at individual cg site can related not only with the regional context, but also with neighboring sites [34]. Therefore, we analyzed the correlation between FXYD family genes expression levels and methylation levels of their promoter cg sites. We found that the expression of FXYD family genes was negatively associated with the methylation level, especially of FXYD1, FXYD3, FXYD5 and FXYD6, indicating that abnormal methylation might be one of the important reasons for the abnormal expression of these genes. However, other genetic or epigenetic alterations, such as gene mutations and copy number changes, might also play roles in aberrant expression of the FXYD gene family.

As previously reported, silencing FXYD2 expression in ovarian cancer cells resulted in the inhibition of Na^+^/K^+^-ATPase activity induced by increased sensitivity to cardiac glycosides, which are Na+/K+-ATPase inhibitors [35]. Overexpression of FXYD3 in digestive tract tumors, including esophageal carcinoma and colorectal cancer, promoted tumor progression and an unfavorable prognosis. This overexpression was thought to be correlated with tumor stage and lymph node metastases [14, 15], and high expression of FXYD3 tended to result in an increased incidence of distant metastasis after patients underwent preoperative radiotherapy for rectal cancer.

With limited research on the role of FXYD4 in cancer progression, we noticed that FXYD4 was physiologically expressed in the distal colon and kidney [10]. FXYD4 increased the affinity of transporters for intracellular Na^+^ and enhanced the transport rate of Na^+^/K^+^-ATPase. In contrast, low Na^+^ intake contributed to FXYD4 upregulation at both the mRNA and protein levels [36, 37]. FXYD5 stimulated the invasion and metastasis of breast cancer cells, and patients with high FXYD5 expression suffered from a lower complete remission rate after receiving RT for head and neck cancer [23, 24]. FXYD5 may be an independent predictor of survival for patients with non-small cell lung cancer [22]. FXYD6 was defined as an oncogenic factor in osteosarcoma cells and is regulated by microRNAs, promoting cell proliferation, migration and metastasis [30, 31].

As useful supplements to these limited results, our study showed that FXYD2, FXYD3 and FXYD4 may be independent prognostic factors for recurrence, while FXYD3 and FXYD7 may be prognostic factors for survival based on univariate and multivariate analyses with Cox regression. Although the results of the aforementioned studies are somewhat inconsistent, the TCGA database used in our study is based on a large number of primary cancer samples and matched adjacent samples. Therefore, our conclusions are convincible, providing insights useful further study of FXYD gene family members as potential prognostic biomarkers and novel therapeutic targets in patients with CC.

Hsu IL reported that FXYD2 was transcriptionally regulated by the transcription factor HNF1B, functioning in tumor growth via autophagy-mediated cell death and modulating the affinity of Na^+^/K^+^-ATPase in ovarian cancer [35]. In breast cancer, the SOX9/FXYD3/Src axis is critical for promoting cancer stem cell function and tamoxifen resistance [38]. In cervical cancer, FXYD3 was confirmed to interact with the LINC01503/miR-342-3p/FXYD3 axis, providing promising therapeutic targets [39]. Studies have revealed that Na^+^/K^+^-ATPase is intimately associated with the EMT as well as the TGF-β1 and NF-κB pathways in malignant tumors [12, 13]. In ovarian cancer, the TGF-β-activated SMAD3/SMAD4 complex is recruited to the promoter region of FXYD5 and promotes FXYD5 transcription [21]. In endometrial cancer, FXYD5 was also reported to be a potential biomarker related to the TGF-β1 and NF-κB pathways, resulting in tumor dissemination. FXYD5 was considered to weaken intercellular adhesion through its extracellular O-glycosylated domain [40].

We further investigated the potential molecular mechanisms by which abnormal expression of FXYD family members regulates the carcinogenesis of CC. The PPI network and Pearson’s correlation analysis revealed that FXYD family members were significantly correlated with each other and played an essential role in interacting with Na^+^/K^+^-ATPase subunits α-1,3,4 and β-1,3. The GSEA demonstrated associations between FXYD family gene expression and several signaling pathways involved in cancer progression, pointing to potential mechanisms underlying the carcinogenicity of FXYD. Notably, FXYD2, FXYD3, FXYD4 and FXYD7 were significantly related to oxidative phosphorylation and the citrate cycle, which indicated an interaction with Na+/K+-ATPase. Studies have reported that oxidative phosphorylation and the citrate cycle are intimately involved in colon cancer [41–43]. In addition, all four members were related to pathways in the extracellular matrix, cell adhesion and cell junctions. Moreover, we found that FXYD3 was significantly correlated with the Wnt signaling pathway, indicating that low expression of FXYD3 in CC may activate this pathway, contributing to carcinogenesis. Interestingly, FXYD4 and FXYD7 were associated with the T cell receptor signaling pathway and the FC epsilon RI signaling pathway, respectively. The TIMER and CIBERSORT analyses showed that FXYD family genes had observable correlations with TIICs, providing evidence for an association between FXYD family member expression and the immune microenvironment in CC. Therefore, we believe that FXYD family members may function as regulators of the immune microenvironment in CC, which is worthy of further investigation.

Although our findings may help to understand the expression patterns and prognostic value of the FXYD family members in colon cancer, there are some limitations deserve mentioning. First, we only got the results through bioinformatics and database analysis, further experimental verification is required. Second, our study is lacking of verification using other freely available databases. Consideration should be given to the possible lack of consistency between the results of different data collections. However, our study has its convincing power for its larger sample-based study by TCGA database.

## CONCLUSION

Taken together, our bioinformatics results indicated that several FXYD family members are differentially expressed in CC. FXYD2, FXYD3 and FXYD4 are revealed as independent prognostic factors for recurrence, while FXYD3 and FXYD7 are identified as prognostic factors for survival. The value of FXYD family members as potential clinical biomarkers in CC patients is worthy of further investigation.

## MATERIALS AND METHODS

### Data resources

Target data including gene expression, clinicopathological features and prognosis information of patients with CC were acquired from the public The Cancer Genome Atlas (TCGA) database (https://portal.gdc.cancer.gov). The TCGA platform provides over 20,000 primary cancer samples and matched adjacent tissues of 33 various cancer types. Using the Genomic Data Commons (GDC) Data transfer tool, we obtained gene transcript data with normalization in Fragments Per Kilobase of transcript per Million mapped reads (FPKM) in our analysis. Data from 480 cases of CC and 41 adjacent normal samples were extracted, and the clinical characteristics of the corresponding patients, including age, gender, tumor (T) stage, node (N) stage and metastasis (M) stage, were used in the analyses.

### Expression data of the FXYD family in CC

The expression information of the FXYD gene family in various types of cancer was obtained from the ONCOMINE database, which is a public oncology microarray database that integrates the expression status of genes in diverse tumors and normal samples (https://www.oncomine.org/). The mRNA expression levels of FXYD family members in CC were extracted from the HTSeq FPKM data by Perl 5.32 software. The differential expression of the FXYD family in CC tissues compared to normal tissues was analyzed using the limma package in R software. The results were represented by a heatmap and box plots, which were visualized by the pheatmap and ggpubr packages.

### Relationship between methylation and mRNA expression of FXYD family members in CC

The Illumina Human Methylation 450K data of TCGA-CC samples were downloaded from UCSC Xena platform, an online exploration tool for public and private, multi-omic and clinical/phenotype data (https://xena.ucsc.edu). The file used for annotating the information on cg sites was obtained from the official website (https://support.illumina.com/downloads/~infinium_humanmethylation450_product_files.html). The methylation levels of cg sites in the gene promoter regions of FXYD family members were recognized. Subsequently, we utilized the Pearson correlation to evaluate the association between methylation and expression of FXYD family members in CC by corrplot package in R software.

### Prognostic value evaluation of FXYD family members in CC

Survival was analyzed by using the Kaplan-Meier method. The associations among each of the potential prognostic factors and the differences between the curves were analyzed by log-rank test. Univariate Cox proportional hazards regression analyses were used to assess the correlations of FXYD family member expression and each clinical variable, including age, gender, T stage, N stage, and M stage, on progression-free survival (PFS) and overall survival (OS) time among CC patients. Furthermore, multivariate Cox proportional hazards models were utilized to estimate the independent prognostic factors by controlling for the significant clinical parameters from the univariate analyses. The survival and survminer packages of R software were used for all analyses and the forest plots were conducted by the ggplot package.

### Pearson correlation and protein-protein interaction (PPI) network of FXYD family members

To evaluate whether FXYD family genes were correlated with each other, a Pearson correlation analysis was performed based on the gene expression data from the TCGA database using the corrplot package of R software. A PPI network was constructed to identify the potentially related proteins interacting with the FXYD family by STRING platform (https://string-db.org).

### Gene set enrichment analysis (GSEA)

Gene set enrichment analysis was performed to investigate the potential molecular mechanisms by which FXYD family genes might participate in tumor progression in CC using GESA software version 4.0.1 (http://software.broadinstitute.org/gsea/index.jsp). Annotated gene dataset file c2.cp.kegg.v7.0.symbols.gmt obtained from the MSig database was used as a reference. In the analysis process, the software divided the samples into high expression group and low expression group according to the median value of specific gene expression. We selected the model of “high expression group *vs* low expression group” and a random combination of at least 1,000 permutations for pathway enrichment analysis. A false discovery rate (FDR) <0.05 were the criteria for the identification of the enriched pathways.

### Corrections between tumor immune infiltrating cells (TIICs) and the FXYD family genes

We explored the associations between TIICs and the FXYD family members in CC by the Tumor Immune Estimation Resource (TIMER) platform (https://cistrome.shinyapps.io/timer/) [44]. In the TIMER database, TIICs include B cells, CD4^+^ T cells, CD8^+^ T cells, dendritic cells, macrophages and neutrophils. Furthermore, CIBERSORT was used for assessing the relationship between independent prognostic FXYD family genes (FXYD2, FXYD3, FXYD4, FXYD7) and the subsets of each immune cell types [45]. According to the median value of gene expression, the samples were divided into high expression group and low expression group. The result was visualized by the vioplot package of R software. CIBERSORT is an analytical tool to provide an estimation of the proportions of 22 tumor-infiltrating lymphocyte subsets in a mixed cell population, using gene expression data (https://cibersort.stanford.edu/).

### Statistical analysis

Perl software 5.32 was used to extract and structure the HTSeq FPKM data, methylation data and GESA preparation documents. R 4.0.3 software with specific packages was used to perform analyses of expression profile, Pearson correlation and prognostic value evaluation of the FXYD family in CC. The intergroup comparison of clinicopathologic variables was performed with the chi-square test with SPSS software 20.0 (IBM, Chicago, USA). *P*<0.05 was considered statistically significant.

### Data statement

The data used for bioinformatics analyses in this study are freely available on The Cancer Genome Atlas (TCGA) program website (https://portal.gdc.cancer.gov) and the UCSC Xena platform (https://xena.ucsc.edu). The interpretation and reporting of these data are the sole responsibility of the authors.

## Supplementary Material

Supplementary Tables
