# Supplementary material for: Expression mode and prognostic value of FXYD family members in colon cancer
Source: Aging (Albany NY). 2021 Jul 15;13(14):18404–22. doi: 10.18632/aging.203290 (PMC8351680; doi:10.18632/aging.203290)
Supplement: Supplementary Tables [file aging-13-203290-s001.pdf]

SUPPLEMENTARY TABLES

Supplementary Table 1. The correlation of FXYD1 expression with clinicopathologic characteristics of CC patients.

Variable		Cases (n)	High	Low	*P* value
		455	129	326	
Age	≥60	332	91	241	0.483
	<60	123	38	85	
Gender	Male	241	67	174	0.835
	Female	214	62	152	
T stage	T1/T2	88	18	70	0.086
	T3/T4	367	111	256	
N stage	Negative	267	59	208	<0.001*
	Positive	188	70	118	
M stage	Negative	339	89	250	0.096
	Positive	116	40	76	

*, *P*<0.05.

Supplementary Table 2. The correlation of FXYD2 expression with clinicopathologic characteristics of CC patients.

Variable		Cases (n)	High	Low	*P* value
		455	409	46	
Age	≥60	332	296	36	0.485
	<60	123	113	10	
Gender	Male	241	212	29	0.163
	Female	214	197	17	
T stage	T1/T2	88	71	17	0.003*
	T3/T4	367	338	29	
N stage	Negative	267	236	31	0.269
	Positive	188	173	15	
M stage	Negative	339	303	36	0.597
	Positive	116	106	10	

*, *P*<0.05.

Supplementary Table 3. The correlation of FXYD3 expression with clinicopathologic characteristics of CC patients.

Variable		Cases (n)	High	Low	*P* value
		**455**	**323**	**132**	
Age	≥60	332	235	97	0.908
	<60	123	88	35	
Gender	Male	241	169	72	0.680
	Female	214	154	60	
T stage	T1/T2	88	66	22	0.433
	T3/T4	367	257	110	
N stage	Negative	267	201	66	0.021*
	Positive	188	122	66	
M stage	Negative	339	250	89	0.033*
	Positive	116	73	43	

*, *P*<0.05.

Supplementary Table 4. The correlation of FXYD4 expression with clinicopathologic characteristics of CC patients.

Variable		Cases (n)	High	Low	*P* value
		**455**	**153**	**302**	
Age	≥60	332	114	218	0.655
	<60	123	39	84	
Gender	Male	241	93	148	0.022*
	Female	214	60	154	
T stage	T1/T2	88	29	59	1.000
	T3/T4	367	124	243	
N stage	Negative	267	90	177	1.000
	Positive	188	63	125	
M stage	Negative	339	113	226	0.821
	Positive	116	40	76	

*, *P*<0.05.

Supplementary Table 5. The correlation of FXYD5 expression with clinicopathologic characteristics of CC patients.

Variable		Cases (n)	High	Low	*P* value
		**455**	**171**	**284**	
Age	≥60	332	132	200	0.128
	<60	123	39	84	
Gender	Male	241	93	148	0.698
	Female	214	78	136	
T stage	T1/T2	88	24	64	0.028*
	T3/T4	367	147	220	
N stage	Negative	267	92	175	0.116
	Positive	188	79	109	
M stage	Negative	339	124	215	0.505
	Positive	116	47	69	

*, $P<0.05$.

Supplementary Table 6. The correlation of FXYD6 expression with clinicopathologic characteristics of CC patients.

Variable		Cases (n)	High	Low	*P* value
		**455**	**183**	**272**	
Age	≥60	332	123	209	0.031*
	<60	123	60	63	
Gender	Male	241	93	148	0.503
	Female	214	90	124	
T stage	T1/T2	88	28	60	0.090
	T3/T4	367	155	212	
N stage	Negative	267	90	177	0.001*
	Positive	188	93	95	
M stage	Negative	339	131	208	0.273
	Positive	116	52	64	

*, $P<0.05$.

Supplementary Table 7. The correlation of FXYD7 expression with clinicopathologic characteristics of CC patients.

Variable		Cases (n)	High	Low	*P* value
		**455**	**158**	**297**	
Age	≥60	332	113	219	0.658
	<60	123	45	78	
Gender	Male	241	92	149	0.115
	Female	214	66	148	
T stage	T1/T2	88	29	59	0.803
	T3/T4	367	129	238	
N stage	Negative	267	84	183	0.090
	Positive	188	74	114	
M stage	Negative	339	117	222	0.910
	Positive	116	41	75	
